# Fungal Melanins Differ in Planar Stacking Distances

**DOI:** 10.1371/journal.pone.0030299

**Published:** 2012-02-16

**Authors:** Arturo Casadevall, Antonio Nakouzi, Pier R. Crippa, Melvin Eisner

**Affiliations:** 1 Department of Microbiology and Immunology and the Division of Infectious Diseases of the Albert Einstein College of Medicine, Bronx, New York, United States of America; 2 Department of Environmental Science, University of Parma, Parma, Italy; 3 Department of Physics, University of Houston, Houston, Texas, United States of America; Massachusetts General Hospital, United States of America

## Abstract

Melanins are notoriously difficult to study because they are amorphous, insoluble and often associated with other biological materials. Consequently, there is a dearth of structural techniques to study this enigmatic pigment. Current models of melanin structure envision the stacking of planar structures. X ray diffraction has historically been used to deduce stacking parameters. In this study we used X ray diffraction to analyze melanins derived from *Cryptococcus neoformans*, *Aspergillus niger*, *Wangiella dermatitides* and *Coprinus comatus*. Analysis of melanin in melanized *C. neoformans* encapsulated cells was precluded by the fortuitous finding that the capsular polysaccharide had a diffraction spectrum that was similar to that of isolated melanin. The capsular polysaccharide spectrum was dominated by a broad non-Bragg feature consistent with origin from a repeating structural motif that may arise from inter-molecular interactions and/or possibly gel organization. Hence, we isolated melanin from each fungal species and compared diffraction parameters. The results show that the inferred stacking distances of fungal melanins differ from that reported for synthetic melanin and neuromelanin, occupying intermediate position between these other melanins. These results suggest that all melanins have a fundamental diffracting unit composed of planar graphitic assemblies that can differ in stacking distance. The stacking peak appears to be a distinguishing universal feature of melanins that may be of use in characterizing these enigmatic pigments.

## Introduction

Several fungal species found in different divisions (phyla) within the Kingdom Fungi commonly synthesize melanin pigments [1], [2]. Fungal melanin is a complex molecule that has been associated with a variety of functions including protection against free radical fluxes, heavy metal toxicity and electromagnetic radiation [1], [3], [4]. In addition, fungal melanization is important for pathogenesis because this pigment has been associated with virulence and acquired resistance to antifungal agents [3], [5], [6]. Melanin was recently proposed to function as an energy transducer in fungi promoting the growth of melanized organisms under gamma radiation fluxes [4]. Melanin is often found in the fungal cell wall where it contributes to cellular structural strength.

Fungal melanins are divided into two major categories known as dihydroxyphenylalanine (DOPA)-melanin and dihydroxynaphthalene (DHN)-melanin depending on their biosynthetic pathway [7]. Like all natural melanins, the structure of fungal melanin remains largely unknown because these pigments are amorphous, insoluble, and are often recovered from natural sources intimately associated with other cellular materials. Consequently, various structural studies have involved synthetic melanins derived from oxidized L-DOPA and DHN but the correspondence of these materials to naturally occurring melanins is uncertain.

One promising system for the study of melanin structure is provided by the human pathogenic fungus *Cryptococcus neoformans*. Unlike most other melanotic fungi *C. neoformans* is unable to synthesize melanin from endogenous precursors and it produces only DOPA-melanin when grown on L-DOPA enriched media. Consequently, cryptococcal cells are non-melanized unless provided with the substrate, L-DOPA, for its specific melanin synthetic enzyme laccase. *C. neoformans* has two major advantages for the study of fungal melanin structure. First, the melanin produced is all derived from a chemically defined exogenous substrate added to the fungal culture thus providing the opportunity for metabolic labeling to gain insight into the structure and biochemistry of the pigment. For example, metabolic labeling with ^13^C-DOPA has been used in combination with solid state NMR to show that *C. neoformans* DOPA-melanin is covalently linked to aliphatic compounds [8], [9]. Secondly, acid digestion of melanized cells yields hollow melanin spheres known as ‘ghosts’ that can be used to study the architecture of cell wall melanin [10].

The proposed structure for a eumelanin protomolecule [11]–[14] was deduced from X-ray diffraction data. The protomolecule consists of four imperfectly stacked planar sheets with each sheet consisting of eight indolequinone molecules linked together so that the oxygen atoms lie on the outer edges of each sheet and the nitrogen atoms are located in a porphyrin-like hole at the center of each sheet. The spacing between sheets is 3.45 A, a distance similar to that found for graphitic sheets. The stack of sheets of melanin forms a diffracting structure which is seen as a prominent broad peak easily discernible in the raw data. However, the information leading to the final proposed structure of the sheets required the subtraction of the background incoherent contributions. X-ray diffraction studies carried out on neuromelanins extracted from human brains (13) have shown the presence of a robust peak similar to that found for eumelanins. The position of the peak, however, suggests a difference in the stacking distance of the neuromelanins compared to the eumelanins. In this study we use X-ray diffraction techniques to compare four different types of fungal melanins. Ours results are consistent with the proposal that melanins are composed of planar structures that can differ in stacking distances.

## Materials and Methods

### Cryptococcus neoformans (Phylum Basidiomycota)

*C. neoformans* strain ATCC 24067(serotype D) was obtained from the ATCC (Rockville, MD). Strain H99 (serotype A) was obtained from Dr. John Perfect (Duke University, NC) and strain NIH-191 (serotype C) was obtained from Dr. Ashok Varma (Bethesda, MD). Cells were grown in minimal medium (20 mM MgSO_4_*7H_2_O, 58.8 mM KH_2_PO_4_, 20 mM Glycine, 15 mM Glucose and 6 uM thiamine) with or without 1 mM of L-dopa at 30°C for ten days with shaking at 150 RPM.

### Aspergillus niger (Phylum Ascomycota)

*A. niger* strain J9901 was obtained from the ATCC. Conidia from *A. niger* were grown on Sabouraud dextrose Agar at pH 5.6 (Difco Laboratories, Detroit, MI) for 1 month at 25°C. Conidia were removed from the agar with 0.05% of tween 20 in PBS by gentle scraping and shaking.

### Wangiella dermatitides (Phylum Ascomycota)

*W. dermatatitides* strain 8656 was obtained from the ATCC. The strain was grown in YPD media (2% peptone, 1% Bacto yeast extract and 2% dextrose) at 37°C with shaking for 1 month.

### Coprinus comatus (Phylum Basidiomycota)

These mushrooms **(** ***“inky caps”*** **) naturally** grow along roadsides in the Pacific Northwest. The termination of the mushroom phase is the formation of black-walled spores along the mushroom cap gills followed by a rapid loss of mass by liquefaction (deliquescence) of the mushroom cap into a black inky fluid containing the spores that drop to the ground. Mushroom caps (n = 5) were collected (Discovery Bay, Sequim, Washington) just as they were changing into an inky black fluid. The fluid was stored at room temperature in plastic storage bags and the fluid shipped to New York for further processing.

### Preparation of melanin

Melanin was isolated by standard protocol as described previously [10]. Briefly, melanized cells were centrifuged at 3,000 rpm for 20 minutes and washed with PBS. Cells were resuspended in a mixture of 1 M sorbitol and 0.1 M of sodium Citrate at pH 5.5, then cell wall lysing enzyme (Sigma Chemical-Aldrich, Inc, St. Louis, MO) was added at 10 mg/ml and incubated overnight at 30°C. Next, cells were washed and incubated in 4.0 M guanidine thiocyanate for 12 h at room temperature with gentle shaking. The melanin preparations were washed with PBS and incubated with 1.0 mg/ml of Proteinase K (Roche Molecular Biochemicals, Indianapolis, IN) in reaction buffer (10 mM Tris, 1 mM CaCl_2_, .05% SDS at pH 7.8) for 4 h at 65°C. After the incubation, samples were washed and resuspended in an aqueous/methanol/chloroform mixture (3∶4∶8, respectively) and extracted three times. The aqueous and particulate fractions were boiled in 6 M HCl for 1 h and washed with PBS and dialyzed extensively in water. Final samples were lyophilized to dryness. All washes were done three times and dialysis was against deionized distilled water. The protocol described above was applied to all the fungal strains.

### Capsular polysaccharide isolated by DMSO extraction

Capsular PS was isolated by a modified protocol described previously [15]. Briefly, yeast cells were grown for six days in minimum medium then centrifuged at 6K RPM and washed three times with Milli-Q water. The cell suspension was mixed with DMSO at a ratio of 1∶2, respectively and incubated for 1.0 hr at room temperature. Cells were removed by centrifugation, and the supernatant was then dialyzed against water (change 2–3 times daily) for at least 72 hrs. Then, the sample was lyophilized to dryness.

### X- ray Diffraction

Samples of lyophilized melanin powders were pressed into discs, 1 cm in diameter and 1 mm thick. The samples were scanned using a Phillips diffractometer operating with a 1.54 Angstrom X-ray beam. Scattering intensity was recorded as a function of the scattering angle. The data were converted from angle coordinates to Q scale with Q being the magnitude of the momentum change of the X-ray photon elastically scattered through an angle Θ. The parameter Q is related to the scattering angle by the equation: Q = 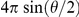)/λ, where λ is the X-ray photon wavelength (1.54 Angstroms in this case). It is useful to relate the Q peak maximum to the distance between the molecular sheets, R, where R = 2π/Q. However, it should be borne in mind that R represents an average value for the distance, the uncertainty being related to the width of the stacking feature in Q space [11]–[14].

### Electron Spin resonance (ESR)

ESR measurement of melanin where done using the same methods as in prior studies [16].

## Results

### Diffraction studies on whole cells

**Figure 1:**
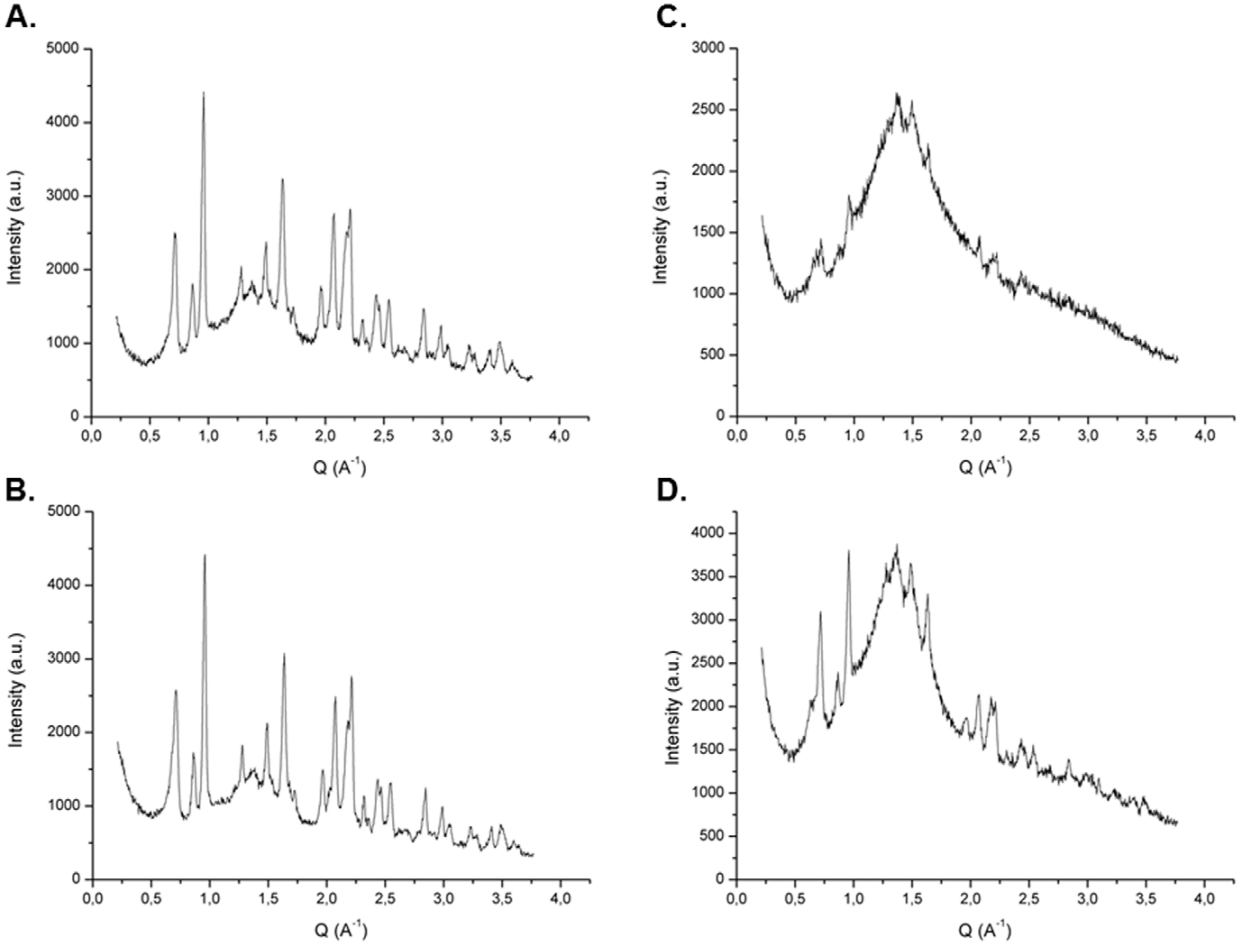
X ray diffraction spectra of whole encapsulated and acapsular cells in their melanized and non-melanized states. Panels: A) Acapsular melanized; B) Acapsular non-melanized; C) encapsulated melanized; and D) encapsulated non-melanized. The spectrum of encapsulated strains is dominated by a broad non-Bragg diffraction feature that is not present in non-encapsulated strains. The sharp Bragg-type diffraction peaks are found in all the spectra and these are likely to originate from microcrystalline elements found in all samples.

**Figure 2:**
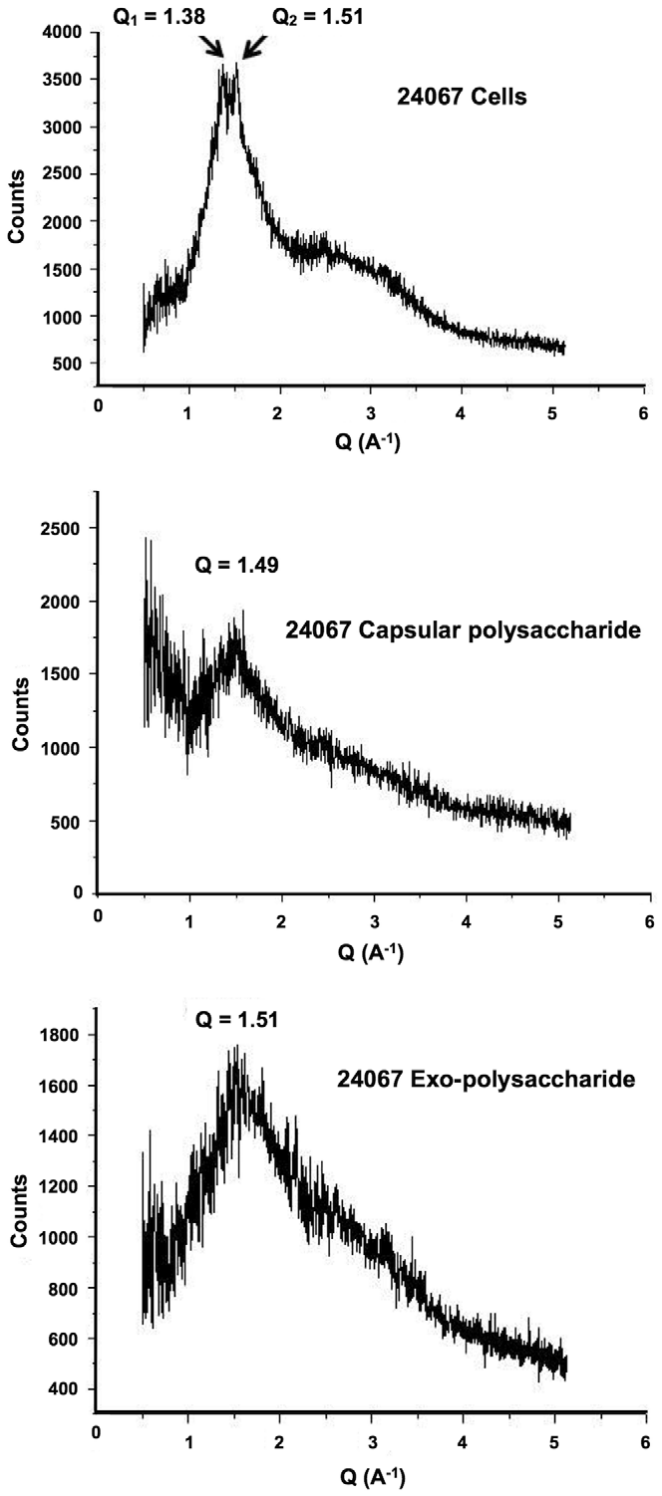
X ray diffraction spectra of 24067 whole encapsulated cells, 24067 exo-polysaccharide and 24067 capsular polysaccharide.

Our initial studies were designed to ascertain whether we could gain insight into melanin structure from pigment in the cell wall. Hence, we compared the X-ray diffraction features observed with melanized and non-melanized whole *C. neoformans* cells using 1.54-Angstrom source. The scattering of X-rays by crystalline structures produces sharp peaks in the diffraction spectrum that serve as a signature for the crystal analyzed. In contrast, amorphous compounds like melanin and polysaccharide produce broad features in the diffraction spectrum known as non-Bragg features resulting from the absence of coherent scattering from regular and repeating structures (e.g., crystals). When whole cells were analyzed the essential feature of these measurements was the presence of a broad non- Bragg feature, whose location was identified in units of Q (reciprocal angstroms). The parameter Q provides a measure of the spacing for the diffracting structure (Figure 1). To explore the origin of this signal we compared the diffraction spectrum of encapsulated and acapsular *C. neoformans* cells in their melanized and non-melanized states (Figure 1). The acapsular strain samples, whether melanized or non-melanized produced a spectrum that lacked the amorphous peak but instead showed several crystalline Bragg peaks (Figure 1). Since the position of these peaks in the melanized and non-melanized strains was the same we conclude that they do not come from melanin. Next we compared the diffraction pattern of whole encapsulated cells, polysaccharide (PS) naturally released from such cells (Exo-PS) and polysaccharide extracted from the capsules (Cap-PS) using dimethethyl sulfoxide (DMSO) from four different cryptococcal strains differing in serotype and polysaccharide structure [Table 1]. Whole cells produced spectra with two Q values, referred to as Q1 and Q2, in the range of 1.38–1.39 and 1.48–1.51, respectively (see Figure 2 for representative spectra). In contrast, the diffraction spectra of isolated exopolysaccharide and polysaccharides released from the capsule was in the range 1.46–1.51, a value consistent with the range for Q2. Numerous Bragg-type diffraction peaks were apparent in all samples studied. Since these peaks occur in the same position irrespective of the presence of capsule or melanin we attribute their origin from microcrystalline impurities. The presence of melanin in the polysaccharide preparation was ruled out by the absence of any pigmentation or ESR signal indicative of a free radical population (data not shown).

**Table 1:** Summary of staking peak for the main non-Bragg diffraction feature observed with cells, exopolysaccharide (Exo-PS) and capsule extracted polysaccharide (CAP-PS) of four strains of *C. neoformans.*

Strain	Serotype	Cells		Exo-PS	CAP-PS
		Q1	Q2	Q	Q
H99	A	1.37	1.48	1.46	1.48
24067	D	1.38	1.51	1.50	1.49
NIH 191	C	1.39	-	-	1.48
NIH 198	B	1.38	1.49	1.51	1.46

**Figure 3:**
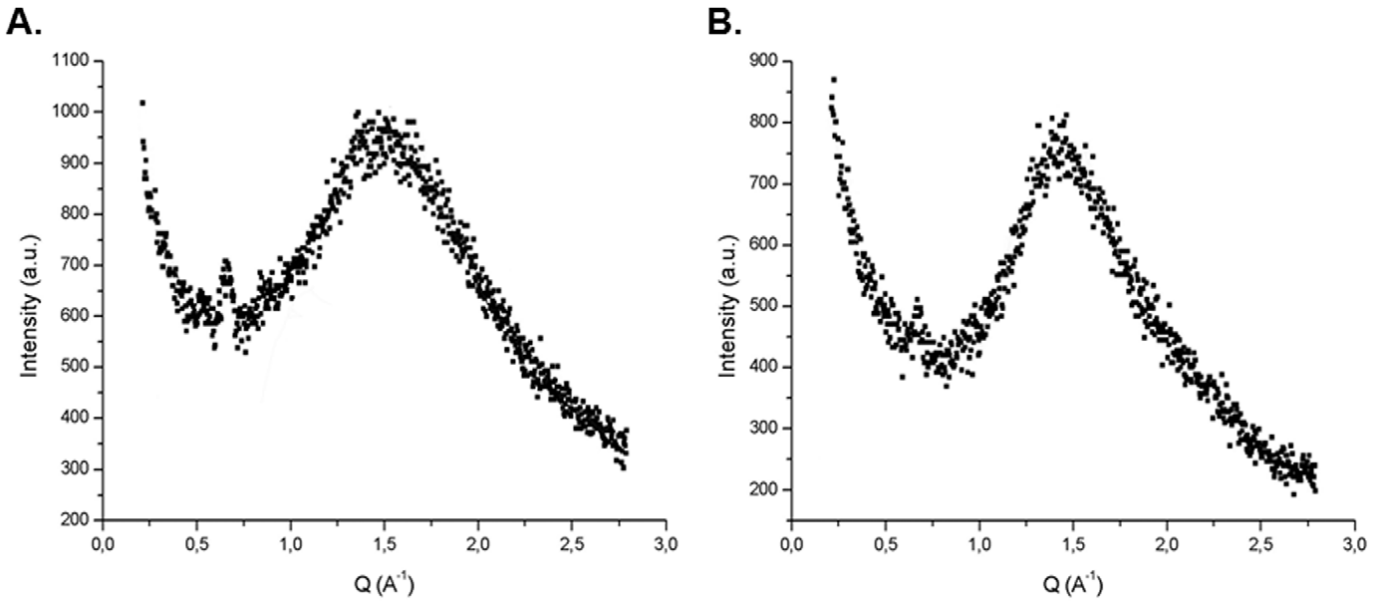
X ray diffraction spectra of melanin isolated from *Wangiella dermatitides* (A) and *C. neoformans* (B). The spectrum of each melanins is dominated by a broad non-Bragg diffraction pattern.

*Fungal melanins*. The results are given as the Q peaks of the X-ray diffraction measurements of the melanins derived from the four different fungal species [Table 2]. The fungal melanins appear to fall into two groups. The melanins of *Aspergillus niger* and *C. neoformans* group into one category with Q peaks of 1.41 and 1.43 and calculated R spacing of 4.39 and 4.45 respectively. The second group was composed of melanin from *Wangiell*a *dermatitides* and the *Coprinus comatus*, which manifested Q peaks of 1.51 and 1.53 corresponding to R spacing of 4.15 and 4.10 angstroms, respectively. Representative spectra are shown in Figure 3.

**Table 2:** Summary of staking peak and distances for various melanins obtained from this study and literature sources.

Melanin Source	Stacking peak Q	Stacking Distance (Å)	Reference
**Fungal melanins**			
*C. neoformans* melanin ghosts	1.43	4.39	This study
*Aspergillus niger*	1.41	4.45	This study
*Wangiella dermatitidis*	1.51	4.15	This study
*Coprinus comatus*	1.53	4.10	This study
**Other melanins**			
Auto oxidized L-dopa	1.64	3.45	[11]
*Sepia officinalis*	1.6	3.46	[11]
Synthetic oxidized dihydroxynapthelene	1.53	4.10	Unpublished
Neuro putamen	1.34	4.67	[14]
Neuro pre-temporal cortex	1.35	4.65	[14]
Neuro cerebellum	1.35	4.65	[14]
Neuro substantia nigra	1.33	4.72	[14]

These values can be compared to those obtained for two synthetic melanins derived from oxidized dihydoxynaphthelene and from oxidized L-Dopa and to eumelanins derived from squid ink (*Sepia officionalis*) and from various pigmented centers in the human brain [Table 2]. The melanins of *Wangiella dermatitides*, *Coprinus comatus* and *Aspergillus niger* have Q peaks and R spacing similar to the synthetic DHN melanin. *C. neoformans* melanin is known to be a DOPA melanin but the Q peak for melanin was smaller than that of autopolymerized L-dopa.

## Discussion

The object of the investigation was to use X-ray diffraction to gain insight into the structure of fungal melanins. Given their amorphous nature melanins are not amenable to study by crystallography and their insolubility precludes the use of solution structural analytic techniques such as NMR. The utility of X-ray diffraction techniques for the analysis of the structure of amorphous materials is limited. The spectra are dominated by the incoherent background that obscures the coherent constructive interference component that carries the structural information. If the atomic composition is known, the incoherent component can be calculated and subtracted. However, in amorphous materials, the orientations of the structural elements are random and the resulting spectrum represents an integrated spatial average. Such diffraction spectra can provide a template for iterative testing of proposed structures by rejecting those structures for which the calculated coherent scattering spectra are in significant disagreement with the measured spectrum after the background is removed.

Melanin pigments are common in the fungal kingdom and their presence has been associated with numerous functions ranging from cellular strength to virulence and energy transduction [2]–[4]. Our initial approach was to study melanin in situ in fungal cell walls by comparing the diffraction from melanized and non-melanized cells grown with and without L-DOPA, respectively. We initially hoped that by using whole cells that we could gain structural insights of melanin in its native cell wall state. Unfortunately, the diffraction spectrum of encapsulated melanized and non-melanized cells was very similar and we could not assign any spectral features to melanin. Consequently, we switched to comparing melanized and non-melanized acapsular cells but again failed to assign melanin spectral features in the diffraction spectrum. Our inability to detect a clear melanin signature from the comparison of melanized and non-melanized cells is perhaps not surprising if one considers that the amount of melanin per cell is likely to be only a small percentage of the total mass [10]. Of interest, we noted the absence of the broad monotonic band in acapsular cells and proceeded to investigate whether this signal was originating from the polysaccharide capsule. Analysis of isolated polysaccharide confirmed that it produced a diffraction spectrum dominated by a broad monotonic band which was intriguingly similar to that of melanin.

To further investigate the nature of the contribution of polysaccharide to the diffraction spectra from whole cells we removed the polysaccharide using DMSO extraction and analyzed it separately. Given the scant information about the conformational structure of cryptococcal polysaccharide we also hoped that the analysis would provide additional information. We noted that for most strains the isolated polysaccharides provided two peaks denoted as Q1 and Q2, suggesting the presence of at least two types of structures in the polysaccharide. Since these values of Q implies an inter-scatter distance that is much larger than any inter atomic distance we infer that it is likely to originate from a repeating structural motif that arises from inter-molecular interactions and/or possibly gel organization. Consequently, we suggest that the broad non-Bragg structural feature arises from an electron dense larger repeating structure that may be altered by organic solvent extraction such as inter-molecular interactions or possibly helical parameters, as have been suggested for this polysaccharide [17]. Although this data does not allow us to propose a model to account for that structure within the context of polysaccharide chemical structures the feature is possibly related to non-covalent associations of polymeric strands, something akin to tangles or knots. Nevertheless, the finding of the capsular non-crystalline feature is interesting and could provide an avenue for new line of investigations to determine the dependence of the feature on experimentally controllable factors.

The detection of signal originating from capsular polysaccharide in the region where the melanin signal was expected suggested that was very difficult to obtain structural insights from cell wall associated melanin by comparing melanized and non-melanized cells. Consequently, we turned our attention to analyzing melanin recovered from fungal cells and comparing their diffraction data to literature studies. Our goal was to analyze *Cryptococcus neoformans* melanin formed when the fungi were grown in a medium which included the melanin precursor l-dopa. Furthermore, we sought to compare *C. neoformans* melanin (DOPA-melanin) to *Wangiella dermatitides* melanin (DHN-melanin). Previous studies had established that melanins presented a diffraction feature whose peak position appeared to vary depending on the process of formation of the various melanins. Although the fungal melanin preparations used exhibit free radical signals characteristic of melanins, these natural melanins almost certainly contain other components, possibly in covalent linkages [8], [9]. The diffraction spectrum revealed that *C. neoformans* melanin produced a peak at approximately Q = 1.4, which is significantly is smaller different from that of polymerized L-Dopa and other eumelanins Q = 1.6 reported previously [11]. Furthermore, diffraction pattern for *Wangiella dermatitidis*, *Aspergillus niger* and *Coprinus comatus* melanins had Q values that differed from both cryptococcal melanin and other eumelanins. The finding that all the fungal melanins yielded Q values suggesting stacking distances in the 4.1–4.5 A range is consistent with the model that melanin from this kingdom also consists of stacked discs that differ in distance based on their composition. In fact, the stacking peak may be a universal feature for melanin that could be used in classifying this enigmatic pigment. Hence, it is conceivable that all melanins share a similar organizational structure but that individual pigments differ in stacking distance details as a result of local differences in composition and possibly associated components, such as the aliphatic material detected in *C. neoformans* melanin by solid NMR analysis [9].

In summary, we conclude that the X-ray diffraction is a useful technique for comparing melanins from various sources and could have usefulness in studying capsular polysaccharides. For instance, we serendipitously observed a diffraction signal from cryptococcal polysaccharide indicative of a repeating structure that could be exploited in future studies to gain new structural insights. Our results provide the first evidence that natural fungal melanins share the basic stacked planar sheet structure proposed for other melanins and indicate that fungal melanins can be identified by their stacking peak parameters and appear to fall into groups subclasses, perhaps related to their biosynthetic pathway differences. Although this alone does not provide information on details of the structure we conclude that the concept of a unique structure for all melanins cannot be supported. While our results are consistent with a general stacking model, it is important to note that were significant differences between the various melanins. More structural information might be obtained by carrying out a systematic analysis of the diffraction spectra including the subtraction of the incoherent background and testing various proposed models against the experimental data. The much simpler task of measuring the location of the stacking peak on many melanins from the raw data could provide some interesting insights into the richness of melanins structure and functions.
